# Increased risk for colorectal cancer under age 50 in racial and ethnic minorities living in the United States

**DOI:** 10.1002/cam4.560

**Published:** 2015-10-16

**Authors:** Rubayat Rahman, Chester Schmaltz, Christian S. Jackson, Eduardo J. Simoes, Jeannette Jackson‐Thompson, Jamal A. Ibdah

**Affiliations:** ^1^ Division of Gastroenterology and HepatologyUniversity of Missouri School of MedicineOne Hospital DriveCE 405ColumbiaMissouri65212; ^2^ Missouri Cancer Registry and Research CenterUniversity of Missouri at Columbia401 Clark HallColumbiaMissouri65211; ^3^ Section of GastroenterologyLoma Linda VA Medical CenterLoma Linda University Medical CenterLoma LindaCalifornia; ^4^ Department of Health Management and InformaticsUniversity of Missouri School of MedicineOne Hospital DriveCE707 CS&E BldgColumbiaMissouri65212

**Keywords:** Alaska natives, American Indian, Asian American, cancer disparities, colorectal cancer, Hispanics, SEER

## Abstract

Colorectal cancer (CRC) is the second most common cause of cancer death in USA. We analyzed CRC disparities in African Americans, Hispanics, Asians/Pacific Islanders, and American Indians/Alaska Natives compared to non‐Hispanic Whites. Current guidelines recommend screening for CRC beginning at age 50. Using SEER (Surveillance, Epidemiology, and End Results) database 1973–2009 and North American Association of Central Cancer Registries (NAACCR) 1995–2009 dataset, we performed frequency and rate analysis on colorectal cancer demographics and incidence based on race/ethnicity. We also used the SEER database to analyze stage, grade, and survival based on race/ethnicity. Utilizing SEER database, the median age of CRC diagnosis is significantly less in Hispanics (66 years), Asians/Pacific Islanders (68 years), American Indians/Alaska Natives (64 years), and African Americans (64 years) compared to non‐Hispanic whites (72 years). Twelve percent of Asians/Pacific Islanders, 15.4% Hispanics, 16.5% American Indians/Alaska Natives, and 11.9% African Americans with CRC are diagnosed at age <50 years compared to only 6.7% in non‐Hispanic Whites (*P* < 0.0001). Minority groups have more advanced stages at diagnosis compared to non‐Hispanic Whites. Trend analysis showed age‐adjusted incidence rates of CRC diagnosed under the age of 50 years have significantly increased in all racial and ethnic groups but are stable in African Americans. These results were confirmed through analysis of NAACCR 1995–2009 dataset covering nearly the entire USA. A significantly higher proportion of minority groups in USA with CRC are diagnosed before age 50 compared to non‐Hispanic Whites, documenting that these minority groups are at higher risk for early CRC. Further studies are needed to identify the causes and risk factors responsible for young onset CRC among minority groups and to develop intervention strategies including earlier CRC screening, among others.

## Introduction

Colorectal cancer (CRC) is the third most common cancer and the second most common cause of cancer death in the United States in both males and females. CRC is a common cause of death among racial and ethnic minorities. For example, African Americans have been reported to have the highest over‐all incidence, highest incidence of advanced stage at disease presentation, highest attributable mortality, and lowest survival rates after diagnosis compared to any other ethnic or racial group 1. Colorectal cancer is the second most common cause of both cancer incidence and cancer death in Hispanics in United States 2. It is the third most common cause of cancer incidence and death in Asian and Pacific Islanders (A/PI) and in American Indians and Alaska Natives (AI/AN) 3. It is important to understand the epidemiology of CRC among racial and ethnic groups, since the Hispanic, A/PI and AI/AN populations are the fastest growing racial and ethnic minority groups in the United States and comprise 23% of total US population 4. It is critical to provide cancer prevention and control programs with the most accurate trends and statistics.

Colorectal cancer incidence and mortality are known to increase with age, however, recent studies showed a significant increase in the CRC onset and incidence in individuals <50 years of age 5, 6, 7, 8, 9. However, little is known about the incidence and survival in various minority and ethnic groups at age <50 years compared to non‐Hispanic Whites (NHW).

The primary aim of the study was to provide a complete and up‐to‐date evaluation of incidence and mortality trends of CRC among racial/ethnic minorities when compared with NHW using SEER (Surveillance, Epidemiology, and End Results) database 1973–2009 and North American Association of Central Cancer Registries (NAACCR) 1995–2009 dataset, including analysis of data in subjects with CRC diagnosed at age <50 years.

## Methods

### Data source and study population

#### SEER database

The SEER program began collecting data on cancer incidence for cases diagnosed in 1973 for seven state and metropolitan registries with an additional two added for 1974 and 1975; these are collectively referred to as the SEER nine Registries. Four more registries began contributing cases diagnosed on and after 1992 (SEER 13). A total of 18 registries (SEER 18) have reported since 2000. Race is categorized in the groupings of NHW, African American, AI/AN, and A/PI for all years. Hispanic ethnicity information is available for all years and is the result of manually collected Hispanic ethnicity and the North American Association of Central Cancer Registries (NAACCR) Hispanic Identification Algorithm (NHIA), which corrects and imputes ethnicity based on last name, birthplace, and other factors 10. Our study population included patients diagnosed with malignant CRC and reported in SEER database from 1973 to 2009, excluding cases diagnosed during the second half of 2005 in Louisiana where data collection was severely impacted by hurricanes Katrina and Rita.

#### NAACCR dataset

NAACCR is the professional organization that certifies population‐based cancer registries in the United States and Canada. We obtained appropriate Institutional Review Board (IRB) approval to receive data on CRC reported to NAACCR by 54 population‐based cancer registries in the United States. The NAACCR Cancer in North America (CINA) Deluxe Dataset contains data from 48 states, the District of Columbia, and five metropolitan areas. This dataset contains data from population‐based cancer registries funded by the Centers for Disease Control and Prevention's (CDC) National Program of Cancer Registries (NPCR) as well as SEER data and contains nearly all the cases of CRC reported in the entire US 11. We used SEER*Stat to calculate age‐adjusted incidence and frequency counts from this dataset. The results were compared to those from the SEER database.

### Statistical analyses

We used SEER*Stat Software 12 to analyze frequency of CRC incidence by descriptive variables such as sex, age, marital status, stage at diagnosis, grade of disease and by race/ethnicity groups. The race/ethnicity groups are NHW, AA, Hispanic (excluding Hispanics in the Alaska Native registry), A/PI, and AI/AN in a Contract Health Service Delivery Area (CHSDA) county 13. In SEER, American Indians and Alaska Natives are reported as a single group and, similar to reports produced by SEER, we only include AI/AN who were diagnosed in a CHSDA county due to evidence that AI/ANs are undercounted by registries in non‐CHSDA counties 13. Age was categorized into <50 years and 50 years or more at diagnosis, with the <50 age group being further categorized as <30, 30–39, and 40–49. Stage was categorized into early (stages I/II) and advanced (stages III/IV) according to AJCC (American Joint Committee on Cancer) 3rd (1988–2003) and 6th (2003–2009) editions reported in SEER. Grade was also compiled into two groups: low (grades I/II) and high (grades III/IV).

We compared the distribution of CRC incident cases of all ages by age‐, gender‐, and cancer‐specific variables such as stage and grade across race/ethnicity groups (NHW, AA, Hispanic, A/PI, and AI/AN). We repeated this analysis for subjects diagnosed with CRC at age <50 years. Missing values were assumed to be missing at random in all groups. Age‐adjusted incidence rates of CRC were calculated based on incidence data from the SEER 13 Registries only from 1992 through 2009.

Population data used to calculate rates in this report was obtained from SEER based on the National Center for Health Statistics (NCHS) bridged single‐race modification to the estimates released by the Census Population Estimates Program; rates were age‐adjusted to the 2000 US Population (Census P25‐1130) standard in 19 groups.

## Results

### Analyses of subjects with CRC diagnosed at any age in SEER database

From 1973 to 2009, approximately 580,000 cases of CRC were diagnosed among NHW in the SEER registries and about 160,000 among racial/ethnic minorities. CRC was the second leading cause of cancer in Hispanics and the third leading cause of cancer in A/PI, AI/AN, AA, and NHW comprising 9.9%, 13.1%, 12.1%, 11.5%, and 11.4% of all cancers in these groups, respectively. Descriptive statistics of CRC cases diagnosed in the above racial/ethnic groups compared to NHW are presented in Table 1. The proportion of men diagnosed with CRC was significantly higher in Hispanics and A/PI compared to NHW, whereas the proportion of women diagnosed with CRC was significantly higher in AA (Table 1). Sex distribution did not differ for AI/AN. Compared to NHW, the percentage diagnosed at an advanced stage, according to AJCC staging schemes, was higher for other racial/ethnic groups.

**Table 1:** Comparison of descriptive statistics of CRC among major US racial/ethnic groups, as reported in SEER database (1973–2009)

Race/Ethnicity	Period	NHW (reference group) *N* = 621,235	AA*N* = 71,309	Hispanic*N* = 45,405	A/PI*N* = 51,762	AI/AN (CHSDA) *N* = 3232
% Male	1973–2009	50.4%	47.9%***	53.2%***	54.0%***	49.5%
	1973–1990	51.2%	48.8%***	54.1%***	55.4%***	49.8%
	1991–2009	50.1%	47.2%***	52.5%**	53.3%**	49.2%
% Married	1973–2009	58.1%	43.1%***	58.0%	67.0%***	57.8%
	1973–1990	61.4%	46.3%***	60.6%	67.6%***	58.5%
	1991–2009	56.5%	41.8%***	57.4%	66.8%***	56.9%
Median age of diagnosis (years)	1973–2009	72	66***	66***	68***	64***
	1973–1990	73	67***	66***	70***	65***
	1991–2009	71	65***	66***	67***	63***
% Age <50 years at diagnosis	1973–2009	6.7%	11.9%***	15.4%***	12.0%***	16.5%***
	1973–1990	5.9%	10.1%***	14.6%***	10.8%***	14.9%***
	1991–2009	7.7%	12.8%***	16.2%***	13.0%***	17.8%***
% Localized SEER summary stage	1973–2009	42.2%	39.3%***	40.4%***	42.0%	38.0%***
	1973–1990	41.3%	38.5%**	39.2%***	41.3%	37.1%***
	1991–2009	43.1%	39.9%***	41.1%**	42.4%	38.7%***
% Regional and distant SEER summary stage	1973–2009	57.8%	60.7%***	59.6%***	58.0%	62.0%***
	1973–1990	58.7%	61.5%***	60.8%**	58.7%	62.9%***
	1991–2009	56.9%	60.1%***	58.9%	57.6%	61.3%***
% AJCC Stage 0 (1988+)	1973–2009	3.0%	3.4%***	3.5%***	3.5%***	1.7%***
% AJCC stages I/II, (1988+)	1973–2009	55.0%	48.4%***	50.7%***	50.9%***	51.6%*
% AJCC stages III/IV (1988+)	1973–2009	42.0%	48.1%***	45.8%***	45.8%***	46.6%***
% Low grades (grade I/II)	1973–2009	78.4%	82.8%***	80.3%***	81.6%***	80.9%*
	1973–1990	77.7%	82.1%***	79.6%**	81.0%***	80.2%**
	1991–2009	79.2%	83.2%***	80.8%**	82.1%***	81.5%***
% High grades (grade III/IV)	1973–2009	21.6%	17.2%***	19.7%***	18.4%***	19.1%*
	1973–1990	22.3%	17.9%***	20.4%**	19%***	19.8%***
	1991–2009	20.8%	16.8%***	19.2%*	17.9%***	18.5%***

NHW: Non‐Hispanic White, AA: African American, A/PI: Asian/Pacific Islander, AI/AN: American Indian and Alaska Native; AJCC: American Joint Committee on Cancer; *P < 0.01, **P < 0.001, ***P < 0.0001, compared with NHW.

We also analyzed the data utilizing a more remote period (1973–1990) and a more modern period (1991–2009); overall, the observed patterns were consistent in both time periods (Table 1).

### Analyses of subjects with CRC diagnosed at age <50 years in SEER database

The median age at diagnosis was significantly lower in all racial/ethnic minority groups compared to NHW (Table 1). Importantly, the percentage of patients diagnosed with CRC at age <50 years in each of the racial/ethnic minority groups was approximately twice that of NHW (Table 1). When the group containing age <50 was further broken down (<30, 30–39 and 40–49), most of the cases with CRC at age <50 years were diagnosed between 40 and 49 years of age. The percentage of Hispanics and A/PI diagnosed in each of the lower two age groups (<30 and 30–39) is significantly higher than NHW (Table 2).

**Table 2:** Comparison of descriptive statistics of CRC among major US racial/ethnic groups diagnosed at age <50 years, as reported in SEER database (1973–2009)

Race/Ethnicity	Period	NHW (reference group) *N* = 38,719	AA*N* = 8470	Hispanic*N* = 6965	A/PI*N* = 5420	AI/AN (CHSDA) *N* = 449
% Male	1973–2009	53.3%	49.0%***	52.9%	52.2%	48.1%*
	1973–1990	51.8%	47.7%***	51.8%	51.4%	47.2%*
	1991–2009	54.9%	50.9%***	53.8%	53.9%	49.0%**
% Married	1973–2009	68.1%	54.2%***	62.9%***	71.7%***	57.2%***
	1973–1990	71.0%	56.8%***	64.2%***	72.8%*	59.5%***
	1991–2009	66.4%	52.6%***	59.8%***	70.6%**	55.3%***
% Age <30	1973–2009	4.8%	4.7%	7.9%***	6.0%***	6.5%
	1973–1990	4.1%	4.4%	7.8%***	6.1%***	6.3%
	1991–2009	5.2%	5.1%	8.0%***	6.0%***	6.6%
% Age 30–39	1973–2009	20.1%	20.9%	26.3%***	23.3%***	24.5%
	1973–1990	19.4%	19.9%	26.1%***	22.8%***	24.2%
	1991–2009	20.3%	21.6%	26.4%***	23.8%***	24.7%
% Age 40–49	1973–2009	75.1%	74.4%	65.8%***	70.7%***	69.%*
	1973–1990	76.5%	76.4%	66.1%***	71.1%***	69.5%*
	1991–2009	74.5%	73.3%	65.6%***	70.2%***	68.7%*
% Localized SEER summary stage	1973–2009	36.4%	33.7%***	34.4%*	34.6%	32.3%
	1973–1990	35.5%	32.3%*	32.0%*	32.9%	30.7%**
	1991–2009	37.2%	35.1%*	37.1%	36.4%	34.1%**
% Regional and distant SEER summary stage	1973–2009	63.6%	66.3%***	65.6%*	65.4%	67.7%
	1973–1990	64.5%	67.7%**	68.0%**	67.1%**	69.3%*
	1991–2009	62.8%	64.9%*	62.9%	63.6%*	65.9%
% AJCC stage 0 (1988+)	1973–2009	2.7%	2.6%	2.6%	2.4%	1.9% (NT)
% AJCC stage I/II(1988+)	1973–2009	45.3%	41.5%***	42.3%***	42.1%***	46.1%
% AJCC stage III/IV (1988+)	1973–2009	52.1%	55.9%***	55.0%***	55.5%***	52.0%
% Low grades (grade I/II)	1973–2009	75.7%	78.5%***	76.2%	74.2%	75.8%
	1973–1990	77.8%	80.3%*	78.3%	76.6%	77.9%
	1991–2009	73.1%	75.5%	75.6%	71.8%	73.2%
% High grades (grade III/IV)	1973–2009	24.3%	21.5%***	23.8%	25.8%	24.2%
	1973–1990	22.2%	19.7%**	21.7%	23.4%	22.1%
	1991–2009	26.9%	24.5%*	23.4%	28.2%**	26.8%

NHW: Non‐Hispanic White, AA: African American, A/PI: Asian and Pacific Islander, AI/AN: American Indian and Alaska Native, NT: not tested due to a small number, *P < 0.01, **P < 0.001, ***P < 0.0001, compared with NHW.

We also analyzed the data at age <50 years utilizing a more remote period (1973–1990) and a more modern period (1991–2009); overall, the observed patterns were consistent in both time periods (Table 2).

**Figure 1:**
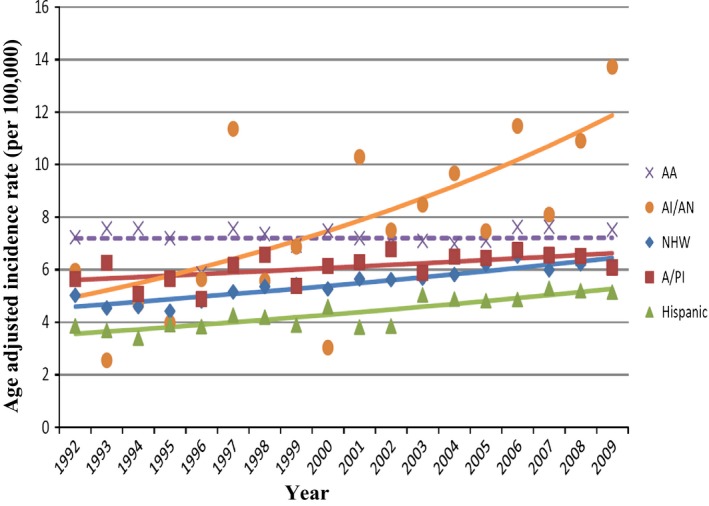
Trend of Age‐Adjusted rate of CRC diagnosed under age 50 by race/ethnicity, SEER 13 Registries (1992+). Points are the observed values. Lines are the fitted values from the joinpoint regression model. Solid lines represent Annual Percent Change which differed significantly from 0 (*P*‐value < 0.05), whereas the dashed line represents insignificant difference from 0. Except among African Americans, all the race/ethnic groups have statistically significant (*P*‐value < 0.05) increase in rates under age 50. Each group differed significantly from the others as determined by pairwise comparisons for coincident trends at alpha = 0.05. AA: African American; AI/AN: American Indian/Alaska Native; NHW: Non‐Hispanic White; A/PI: Asian/Pacific Islander.

The average annual percent change (AAPC) of the age‐adjusted incidence rates for various racial/ethnic groups were calculated according to age groups as reported in the SEER 13 registries for the period 1992–2009 (Table 3). For all ages at diagnosis, the age‐adjusted incidence rates for NHW, AA, and A/PI have significantly decreased (AAPC < 0, Table 3). In contrast, for ages <50 years at diagnosis, the age‐adjusted incidence rates were noted to increase significantly in each of the racial/ethnic groups except in AA (AAPC > 0, Table 3). Figure 1 shows the trend of CRC incidence in this younger age group (<50 years) over the period 1992–2009; the age‐adjusted incidence rates have increased significantly in each of the racial/ethnic groups except AA. Although the data for AI/AN are scattered due to the low number of AI/AN analyzed compared to other minority groups, the upward trend of the incidence rate in young AI/AN is significant (Fig. 1).

**Table 3:** Average Annual Percent Change (AAPC) of the age‐adjusted CRC incidence rates in major US racial/ethnic groups according to age groups, as reported in the SEER 13 registries (1992–2009)

Race/Ethnicity	AAPC		
	All Ages	Age ≥50 years at diagnosis	Age <50 years at diagnosis
All	−1.61*	−1.86*	1.68*
NHW	−1.81*	−2.09*	2.02*
AA	−0.95*	−1.05	0.01
Hispanics	−0.20	−0.41	2.35*
A/PI	−1.15*	−1.32*	0.98*
AI/AN (CHSDA)	−0.39	−1.07	5.29*

NHW: Non‐Hispanic White, AA: African American, A/PI: Asian and Pacific Islander, AI/AN: American Indian and Alaska Native; *P < 0.05. APC tested if different than 1.

### Survival of CRC

Colorectal cancer survival for all ages during the study period showed that the 1‐ and 5‐year relative survival rates for Hispanics and A/PI were significantly higher in comparison to NHW but lower for AA when compared with NHW. The 5‐year relative survival was lower for AI/AN than NHW (Table 4). In contrast, for age <50, the 5‐year relative survival rates for AA and Hispanics were significantly lower than NHW. There was no difference in the 5‐year relative survival rates when A/PI and AI/AN were compared to NHW.

**Table 4:** CRC survival in major US racial/ethnic groups, as reported in SEER database (1973–2009)

Relative Survival, %	Non‐Hispanic White (Reference Group)	African American	Hispanic	A/PI	AI/AN (CHSDA)
Diagnosed, all ages					
*N*	462,402	58,703	38,377	39,106	2319
1 year	80.8%	77.9%***	83.3%***	85.5%***	80.0%
5 years	61.1%	53.8%***	61.4%***	63.5%***	54.9%*
Diagnosed at age <50 years					
*N*	35,562	7803	6363	5025	409
1 year	88.3%	85.1%***	88.4%	90.2%***	84.6%
5 years	65.6%	56.4%***	62.0%***	65.9%	59.8%

A/PI: Asian and Pacific Islander, AI/AN: American Indian and Alaska Native; *P < 0.01, ***P < 0.0001, compared with NHW.

### Analysis utilizing NAACCR dataset 1995–2009

Analysis of NAACCR CINA Deluxe Dataset 1995–2009, using SEER*Stat, showed that the calculated age‐adjusted incidence rate of CRC from NAACCR CINA Deluxe database was comparable to the age‐adjusted incidence rate of CRC using SEER 13 database 1992–2009 according to major US racial/ethnic groups. Additionally, the CINA data also showed increasing incidence rates among people under age 50 despite overall decreases for all ages combined. The median age of diagnosis and the proportion of CRC cases diagnosed under age 50 were also comparable between these two databases (Table 5).

**Table 5:** Comparison of CRC demographics between NAACCR CINA Deluxe database 1995–2009 and SEER database 1973–2009 according to major US racial/ethnic groups

Variables Database	Age‐adjusted incidence rate per 100,000		Median age of diagnosis (years)		Age <50 years at diagnosis (%)	
	SEER 13 Registries (1992+)	NAACCR1995–2009	SEER1973–2009	NAACCR1995–2009	SEER1973–2009	NAACCR1995–2009
NHW (Reference group)	51.5	51.4	72	70–74	6.7%	7.2%
AA	61.0***	58.6***	66***	65–69***	11.9%***	12.7%***
Hispanic	38.9***	42.0***	66***	65–69***	15.4%***	15.2%***
A/PI	44.5***	38.8***.	68***	65–69***	12.0%***	14.1%***
AI/AN (CHSDA)	52.5	N/A	64***	N/A	16.5%***	N/A
AI/AN (All counties)		34.6***		60–64***		15.7%***

NHW: Non‐Hispanic White, AA: African American, A/PI: Asian and Pacific Islander, AI/AN: American Indian and Alaska Native; NAACCR: North American Association of Central cancer Registries; SEER: Surveillance, Epidemiology, and End Results; ***P < 0.0001, compared with NHW.

## Discussion

There were two major findings in our study. Racial/ethnic minority groups are diagnosed with CRC at a significantly earlier age and are diagnosed with CRC at more advanced stages when compared with NHW. The proportion of cases diagnosed with CRC at age <50 in minority groups were approximately twice that of NHW (1.8‐, 2.3‐, 1.8‐, and 2.5‐fold for AA, Hispanics, A/PI, and AI/AN, respectively). Analysis utilizing all ages revealed that minority groups were diagnosed with CRC at more advanced stages compared to NHW (Table 1). At ages <50, a significantly higher proportion of AA, Hispanics, and A/PI were diagnosed with CRC at advanced stages (III/IV) compared to NHW (Table 2).

There are several potential factors that influence development of CRC at age <50 years including hereditary factors, environmental factors, and diet/life style factors that may lead to epigenetic changes 14, 15, 16, 17, 18, 19, 20, 21. Hereditary factors and family history may play an important role in the increased incidence of CRC diagnosed before age 50 in minority populations. The understanding of family history of CRC is not as well known among minority groups as it is among NHW. For example, Hispanics are less likely to know about a family history of CRC when compared with NHW 16. This may be true among AA, but the data are conflicting 17, 18. The same may be true among the A/PI population 19, 20. The lack of understanding of family history may translate into patients from certain minority populations not undergoing CRC screening at the appropriate time.

Reasons for diagnosis of advanced stage CRC among racial/ethnic minorities have been noted to be multifactorial. Racial/ethnic minorities continue to have lower CRC screening rates than NHW even though national increases in CRC screening have occurred 22, 23, 24. CRC screening rates among NHW were reported to be substantially higher than among AA, Hispanics, A/PI, and AI/AN even in equal access centers 25, 26, 27. An evaluation of the National Health Interview Survey data found that race, age, education, income level, and having a usual source of health care, and insurance were associated with up‐to‐date CRC screening 24. The causes for AI/AN under‐screening by an endoscopic procedure include a lack of endoscopic services at most Indian Health Service (IHS) and tribal facilities, and underfunded referral systems 28. Of increasing concern is the disparity between AI/AN who live in Alaska and NHW; between 2005 and 2009, AI/AN persons in Alaska had a 115% greater CRC incidence rate and a 132% greater death rate than NHW 28.

Regular screening for CRC is the essential key for prevention and early diagnosis of colorectal cancer. The U.S. Preventive Services Task Force (USPSTF) recommends screening for CRC using FOBT, sigmoidoscopy, or colonoscopy beginning at age 50 years; people at higher risk of developing CRC, such as those with family history of CRC, should begin screening at a younger age and may need to be tested more frequently 29. According to recommendations from the American College of Gastroenterology published in 2009, African Americans should begin screening for CRC at age 45 years rather than 50 years because of increased incidence of CRC in this group before 50 years of age 1, 30. Our analysis is consistent with this recommendation, indicating that 11.9% of AA compared to 6.7% of NHW CRC cases develop prior to 50 years of age. Our findings of early, more advanced CRC development in other racial/ethnic groups, and of increasing age‐adjusted incidence rates before age 50, is alarming and indicates a need for examination of current guidelines for CRC screening for all minority groups living in the United States.

The SEER Program collects and publishes cancer incidence and survival data from population‐based cancer registries that currently cover more than 28% of the US population, providing a large high‐quality database for assessing differences among racial/ethnic groups. The population covered by SEER is comparable to the general US population; however, it tends to be somewhat more urban and has a higher proportion of foreign‐born persons than the general US population 31. A strength of our study is that our conclusions utilizing SEER database analysis are validated using NAACCR dataset 1995–2009, which covers nearly the entire US population. The age‐adjusted incidence rates of CRC, median age of diagnosis, and proportion of cases diagnosed at age <50 are fairly similar except for the rates for AI/AN, which are not strictly comparable due to the lack of a county variable that would allow only those AI/AN living in a CHSDA county to be included.

In summary, we analyzed disparities of CRC among racial/ethnic groups at a national level utilizing large population‐based data extracted from the SEER database with confirmation of results utilizing the NAACCR CINA data virtually covering the entire United States. Large disparities continue to persist among racial/ethnic minorities compared to NHW. Minority groups are at higher risk for early CRC. These findings suggest a need for further studies to address this alarming trend and examine the causes and risk factors involved. In addition, studies are needed to develop and test intervention strategies including a consideration for lowering the screening age in minority groups, among other strategies.

## Conflict of Interest

None declared.
